# Endoscopic Ultrasound-Guided Variceal Therapy for Gastric Variceal Rupture After Failed Endoscopic Injection Sclerotherapy: A Case Report

**DOI:** 10.7759/cureus.84354

**Published:** 2025-05-18

**Authors:** Sakue Masuda, Atsushi Irisawa, Jun Kubota, Masahiro Kobayashi, Kazuya Koizumi

**Affiliations:** 1 Department of Gastroenterology Medicine Center, Shonan Kamakura General Hospital, Kamakura, JPN; 2 Department of Gastroenterology, Dokkyo Medical University, Mibu, JPN

**Keywords:** case report, cyanoacrylate injection, endoscopic ultrasound, gastric varices, interventional eus, variceal bleeding

## Abstract

Endoscopic ultrasound (EUS) has become an essential tool in vascular interventions due to its imaging potential in the proximity of various vascular structures in the mediastinum and abdominal cavity. EUS-guided variceal therapy (EUS-VT) has emerged as a promising approach for treating gastric varices (GV). Although still underused, EUS-VT offers such advantages as precise targeting and real-time variceal puncture visualization, making it a valuable alternative when conventional therapies fail. We report the case of a 63-year-old man with GVs secondary to fatty liver cirrhosis, who experienced repeated variceal ruptures despite multiple prior interventions (e.g., balloon-occluded retrograde transvenous obliteration, percutaneous transhepatic obliteration, partial splenic embolization, and several endoscopic injection sclerotherapy (EIS) sessions). During his most recent variceal rupture episode, EUS-VT was performed. A 23-gauge sclerotherapy needle failed to reach the target varix under EUS guidance. However, successful puncture and obliteration could be achieved using a 22-gauge fine-needle aspiration needle and cyanoacrylate glue. Doppler imaging confirmed lipiodol distribution and the absence of blood flow, indicating successful embolization. No adverse events occurred or rebleeding was observed during the one-month follow-up. This case demonstrates the clinical utility of EUS-VT as a rescue therapy for gastric variceal bleeding in patients with anatomically complex lesions refractory to standard treatments. EUS-VT should be considered a viable option in selected cases when conventional EIS is unsuccessful due to anatomical limitations.

## Introduction

Due to its imaging potential in the proximity of various vascular structures in the mediastinum and abdominal cavity, endoscopic ultrasound (EUS) has become an essential tool for vascular interventions [1]. EUS-guided vascular therapy is reportedly highly useful in cases of variceal bleeding [1-3]. EUS guidance-assisted gastric variceal treatment is referred to as EUS-guided variceal therapy (EUS-VT).

EUS-VT is a technique in which target vessels are visualized and treated by injecting or deploying agents such as cyanoacrylate (CYA)-based adhesives, sclerosing agents, or embolization coils. In contrast to conventional endoscopy, where only exposed bleeding vessels can be directly visualized and treated, EUS allows for direct therapeutic intervention in vascular lesions located within the submucosa or deeper layers, which are otherwise inaccessible to direct endoscopic view. Due to its ability to directly access target vessels under ultrasound guidance, EUS-VT may be a viable option even in cases where anatomical challenges preclude the use of conventional approaches such as PTO or BRTO. The first report of EUS-guided CYA injection targeting the feeder of isolated gastric varices (GV) in 2007 described obliteration in all five treated cases [4]. In 2010, the same group reported the first EUS-guided coil deployment case for isolated GV [5].

Conventional endoscopic injection sclerotherapy (EIS) displays approximate technical success, complication, and long-term rebleeding rates of 95% and 30% and 23-40% of the cases, respectively [6]. Although historical comparisons suggest that EUS-VT retains a similar technical success with lower complication and rebleeding rates [7], this approach has not been widely adopted as a standard treatment and is typically conducted only under ethics committee approval at individual institutions. Here, we present a case in which EUS-VT successfully yielded hemostasis in a patient with gastric variceal rupture upon unsuccessful EIS.

## Case presentation

A 63-year-old man with a 10-year history of liver cirrhosis developed GV, detected during routine outpatient follow-up. The patient had compensated cirrhosis due to non-alcoholic fatty liver disease, with a Child-Pugh score of B. Although there was no history of overt hepatic encephalopathy, persistent hyperammonemia was observed.The varices ruptured repeatedly over several years. He had undergone multiple treatments, including balloon-occluded retrograde transvenous obliteration (BRTO), percutaneous transhepatic obliteration (PTO), partial splenic embolization (PSE), and multiple EIS sessions. One year after the last intervention, he presented again with a gastric variceal rupture.

**Figure 1:**
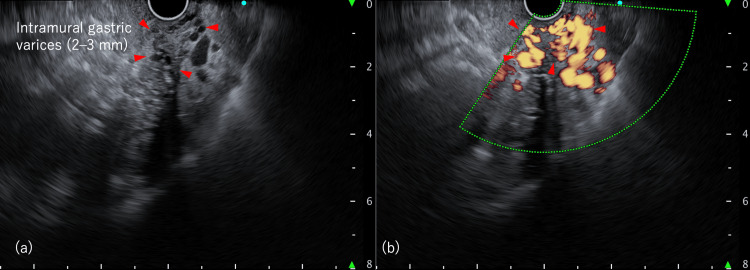
Endoscopic ultrasound images of gastric varices (a) B-mode image: tortuous intramural gastric varices (2–3 mm) and an extramural varix (4 mm). (b) Color Doppler mode image. The blood flow within the gastric varices is clearly visualized. Red arrowhead: gastric varices.

Due to the failure of previous EIS attempts and the presence of tortuous, non-protruding varices not amenable to direct endoscopic treatment, EUS-VT was considered the most feasible rescue approach. EUS-VT was performed using a convex-arrayed EUS (GF-UCT 260; Olympus Corp., Tokyo, Japan). The splenic vein was visualized from within the stomach to identify GV continuously from the feeder vessels. After confirming the target GV, the maximum diameter was measured at 2-3 mm (Figure 1).

Prior to fine-needle aspiration (FNA) needle application, a 23-gauge needle designed for sclerotherapy of varices (Varixer; TOP Corp., Japan) was attempted. However, under EUS guidance, the Varixer needle could not reach the target GV or adequately penetrate the wall. Therefore, a 22-gauge EUS-guided FNA needle (EZ Shot 3 Plus; Olympus Corp.) was used instead. The varix was punctured, and a mixture of 1.5 mL of cyanoacrylate glue (CYA) and 0.8 mL of Lipiodol was injected.

**Figure 2:**
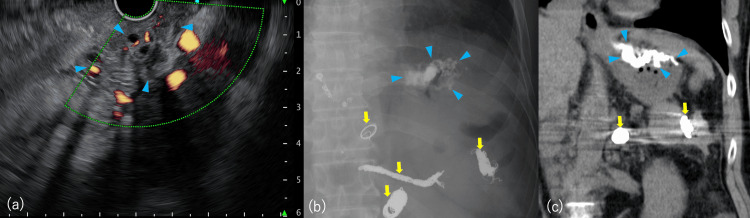
Post-treatment results of endoscopic ultrasound (EUS)-guided vascular therapy (a) Color Doppler endoscopic ultrasound. The previously observed blood flow within the gastric varices disappeared after the treatment. Blue arrowhead: gastric varix with absent blood flow. (b) Fluoroscopic image revealing lipiodol distribution within the varices. Blue arrowhead: gastric varix filled with lipiodol. Yellow arrow: coils and embolization plugs placed during prior procedures, including balloon-occluded retrograde transvenous obliteration (BRTO), percutaneous transhepatic obliteration (PTO), and partial splenic embolization (PSE). (c) Computed tomography image presenting lipiodol distribution. Blue arrowhead: gastric varix filled with lipiodol. Yellow arrow: coils and embolization plugs placed during the previous BRTO and PSE procedures.

Lipiodol distribution within the GV was confirmed. Color Doppler EUS demonstrated blood flow absence, indicating successful obliteration (Figure 2). The procedure was completed without adverse events or rebleeding during the one-month follow-up after the procedure.

A timeline of the patient's gastric variceal treatments is presented in Table 1.

**Table 1:** Timeline of gastric variceal treatments

Date	Treatment	Outcome/note
Apr 2021	Partial splenic embolization (PSE)	Performed to reduce portal pressure
Dec 2021	Percutaneous transhepatic obliteration (PTO)	Varices persisted
Jul 2022	Balloon-occluded retrograde transvenous obliteration (BRTO)	Varices persisted
Sep 2022	Endoscopic injection sclerotherapy (EIS)	Partial effect
Nov 2022	EIS	Recurrence
Dec 2022	EIS	Recurrence
Feb 2024	EIS	Recurrence
Mar 2024	EIS	Recurrence
Apr 2025	EIS (final attempt)	Failed to access the varix
Apr 2025	EUS-guided variceal therapy (EUS-VT)	Successful hemostasis achieved

## Discussion

This case presents successful EUS-VT application in a patient with GV rupture refractory to multiple prior therapies, including BRTO, PTO, PSE, and multiple EIS sessions. The complex morphology of the repeatedly treated varices precluded effective treatment using conventional techniques. EUS guidance allowed for real-time visualization and precise puncture, even in the case of small (2-3 mm) varices.

EUS offers both theoretical and practical advantages over conventional techniques [1] as follows: 1) identification of variceal size and number for precise therapy; 2) feeder vessel, perforator, or shunt detection; 3) real-time variceal puncture visualization; 4) elimination of direct endoscopic visualization dependence, particularly beneficial during active bleeding or when gastric contents obstruct the view; and 5) immediate obliteration confirmation through Doppler assessment.

McCarty et al. conducted a meta-analysis comparing EUS-guided coil monotherapy, cyanoacrylate (CYA) monotherapy, and combined coil plus CYA therapy for the treatment of GV. They reported that the combined coil and CYA approach showed superior efficacy and safety compared to either modality alone. Specifically, treatment efficacy was 98% with coil plus CYA versus 96% with CYA monotherapy (p < 0.001), and the adverse event rate was 10% versus 21%, respectively (p < 0.001). When compared to coil monotherapy, efficacy was 96% versus 90% (p < 0.001), while adverse event rates were 10% versus 3%, respectively (p = 0.057) [8]. Although certain reports suggest combining CYA with coils may improve safety [8-10], in this case, coil deployment was deemed difficult due to the narrow diameter and complex anatomy of the varices. Therefore, the treatment was successfully performed with CYA alone. While EUS-VT remains underutilized, this case highlights its clinical utility in managing GV when endoscopic injection sclerotherapy proves to be technically or anatomically challenging.

In this case, we also encountered specific technical challenges during EUS-VT. Although the needle tip appeared to be positioned within the varix on EUS imaging, it was initially pressing against the flexible wall without actual penetration. Even when the needle seemed to be inside the varix, the absence of blood backflow made it difficult to determine whether the needle had failed to penetrate the variceal wall or was merely in contact with the inner wall of the narrow-caliber varix (2-3 mm in diameter). To confirm intravariceal placement, we cautiously advanced the needle further. A subtle “give” sensation was felt, accompanied by observable tissue movement on the EUS image. Shortly thereafter, a small amount of blood return was confirmed, indicating successful needle placement. This experience highlights that, in cases involving small varices, visual confirmation on EUS alone may be insufficient, and careful tactile feedback combined with dynamic imaging observation is essential to ensure accurate needle positioning.

This case report has several limitations. First, the technical success of the procedure was assessed using Doppler ultrasound findings and plain CT imaging to confirm Lipiodol distribution. However, objective post-treatment measures such as changes in variceal size were not obtained. Due to multiple prior interventions, the varices lacked prominent luminal protrusion, making endoscopic size assessment impractical. In addition, post-procedural CT evaluation was limited because the varices had been cast with cyanoacrylate (CYA), which precluded short-term size comparisons typically possible after ethanolamine oleate with iopamidol treatment. A follow-up CT scan is planned at six months post-procedure to allow more objective assessment of long-term variceal regression. Second, the follow-up period at the time of reporting was only one month. Although no rebleeding was observed during this time, this short interval is insufficient to evaluate long-term outcomes such as rebleeding risk or variceal recurrence. These limitations should be considered when interpreting the clinical utility of EUS-guided variceal therapy in this case. Despite these limitations, the clinical insights from this case remain valuable and contribute to the growing evidence supporting EUS-VT as a feasible intervention in anatomically difficult scenarios.

This case illustrates not only the feasibility but also the technical nuances of EUS-VT in anatomically challenging scenarios. Given its advantages and growing evidence base, EUS-VT should be considered a valuable rescue option when conventional therapies such as EIS are unsuccessful.

## Conclusions

This case underscores the significant clinical relevance of EUS-VT in managing gastric variceal rupture, particularly when conventional EIS fails due to anatomical challenges. The potential of EUS to visualize feeder vessels and varices in real time enabled precise targeting and successful hemostasis in a patient with complex variceal anatomy that rendered EIS ineffective. Therefore, EUS-VT should be considered a viable rescue option in anatomically refractory cases of variceal bleeding.
